# Computational prediction of new auxetic materials

**DOI:** 10.1038/s41467-017-00399-6

**Published:** 2017-08-22

**Authors:** John Dagdelen, Joseph Montoya, Maarten de Jong, Kristin Persson

**Affiliations:** 1 Lawrence Berkeley National Laboratory, 1 Cyclotron Rd, Berkeley, CA 94720 USA; 2 SpaceX, 1 Rocket Road, Hawthorne, CA 90250 USA; 3 University of California, Berkeley, 210 Hearst Memorial Mining Building, Berkeley, CA 94720 USA

## Abstract

Auxetics comprise a rare family of materials that manifest negative Poisson’s ratio, which causes an expansion instead of contraction under tension. Most known homogeneously auxetic materials are porous foams or artificial macrostructures and there are few examples of inorganic materials that exhibit this behavior as polycrystalline solids. It is now possible to accelerate the discovery of materials with target properties, such as auxetics, using high-throughput computations, open databases, and efficient search algorithms. Candidates exhibiting features correlating with auxetic behavior were chosen from the set of more than 67 000 materials in the Materials Project database. Poisson’s ratios were derived from the calculated elastic tensor of each material in this reduced set of compounds. We report that this strategy results in the prediction of three previously unidentified homogeneously auxetic materials as well as a number of compounds with a near-zero homogeneous Poisson’s ratio, which are here denoted “anepirretic materials”.

## Abstract (web summary)

There are very few inorganic materials with auxetic homogenous Poisson’s ratio in polycrystalline form. Here authors develop an approach to screening materials databases for target properties such as negative Poisson’s ratio by using stability and structural motifs to predict new instances of homogenous auxetic behavior as well as a number of materials with near-zero Poisson’s ratio.

## Introduction

A material’s Poisson’s ratio reflects the deformation of its cross-section in response to an orthogonal tensile strain. The term “auxetic” defines a class of exotic materials that exhibit a negative Poisson’s ratio, which causes a counter-intuitive expansion under tension rather than contraction^1^. These materials have been shown to possess enhanced hardness and toughness, as well as absorb vibrations and sound better than their non-auxetic counterparts^2, 3^. As a result, the atypical elastic behavior of auxetic materials is enabling advancements in a broad range of technologies such as impact-resistant composites, extremely precise sensors, tougher ceramics, and high-performance armor^2–5^.

**Fig. 1:**
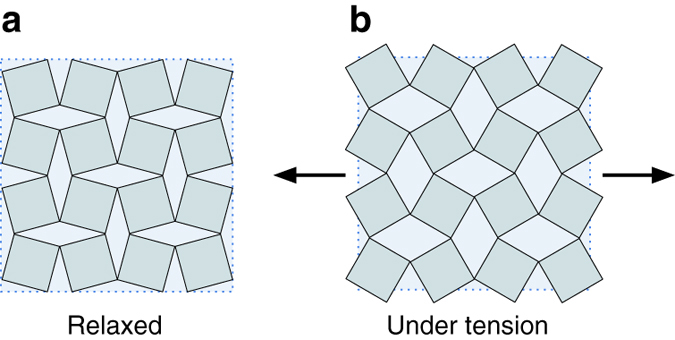
The “rotating squares” model for auxetic behavior. **a** The relaxed structure under no strain and **b** the structure under uniaxial tension showing positive strain along both axes

Auxetic behavior is generally believed to originate at the structural level. Materials with anisotropic mechanical behavior, such as crystalline materials, exhibit orientation-dependent directional Poisson’s ratios, *ν*_*ij*_ (where *i* and *j* are the directions of the applied tensile strain and the resultant transverse strain). For polycrystalline solids, which are macroscopically isotropic, these directional values average to a “homogeneous” value, *μ* ^2^. A number of crystalline materials have been found to exhibit negative Poisson’s ratios in certain directions associated with specific features of their crystal structures^6–9^. However, nearly all of the currently known homogeneously auxetic materials are porous foams or purposefully designed hinged meta-materials with open, re-entrant structures^3, 10, 11^. The most well-known example of a crystalline material with a negative homogeneous Poisson’s ratio, *α*-cristobalite (SiO_2_), was discovered via laser Brillouin spectroscopy in 1992 by Yeganeh-Haeri et al.^12, 13^. They hypothesized that the material’s unusual elastic behavior originates from the rotation of rigid SiO_4_ tetrahedra. Indeed, the prevailing theory for auxetic behavior in *α*-cristobalite and other inorganic materials relies on rigid unit modes (RUM’s) in which connected polyhedra rotate without deforming (see Fig. 1)^2, 3, 14–17^. Interestingly, the unique deformation behavior of this class of materials may also induce specific phase transformations. For example, at high temperature, *α*-cristobalite transforms into *β*-cristobalite where the structural transformation path is characterized by a static rotation of the silica tetrahedra^18, 19^.

Some zeolites with RUM features have been predicted as auxetic using semi-empirical molecular force field models^20, 21^; however, subsequent experiments were only able to verify negative Poisson’s ratio for certain directions, not homogeneous auxetic behavior. Goldstein et al. report negative average Poisson’s ratios for a small number of tetragonal and cubic materials calculated from previously published elastic constants^22, 23^. These include C_16_ FeGe_2_, elemental Ba, and solid solutions based on rocksalt-structured SmS. However, we note that other reference work cites the Poisson’s ratio of Ba as 0.28^24^ and the calculated Poisson’s ratio of C_16_ FeGe_2_ is obtained as 0.24^25^. Previous studies have also found unusual Poisson’s ratios to correlate with elastic anisotropy^26, 27^. In fact, auxetic behavior in certain directions is not uncommon for materials with some degree of anisotropy, such as cubic metals^6^ and layered materials^2, 26, 28^. However, it is worth emphasizing that the existence of auxetic directions alone is not enough to render a material’s bulk, average Poisson’s ratio negative. Finally, we note that—with the possible exceptions listed above—no new homogeneously auxetic crystalline materials have been discovered since *α*-cristobalite SiO_2_.

In this work, we utilize a screening process for auxetic materials that employs open-source tools and algorithms to efficiently and systematically find promising candidates. Several open materials databases with structural information are available today^29, 30^; for this study, we use the Materials Project^31^, which provides access to structure information for more than 67 000 materials and open-source structure manipulation software^32^. This process yields 38 candidate materials, which are subsequently investigated via first-principles density functional theory (DFT) calculations of their full elastic tensors. Of the 38 candidates, 7 new compounds are identified to exhibit negative homogeneous Poisson’s ratios in addition to the known auxetic *α*-cristobalite. The most notable of these predictions is a high temperature aluminum orthophosphate polymorph, HT-AlPO_4_. The other new materials predicted to exhibit auxetic behavior are four hypothetical SiO_2_ polymorphs and two hypothetical metal vanadate structures. Additionally, we report near-zero homogeneous Poisson’s ratios for a number of materials in various chemical systems, which we denote “anepirretic materials” after the Greek word for “unaffected.”

## Results

### Screening process

**Fig 2:**
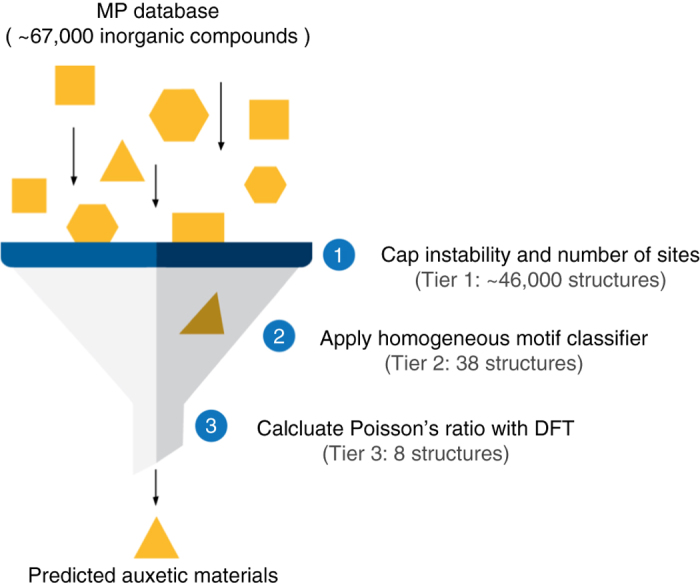
Overview of the screening process. Tier I filtered on formation energy and number of sites, thereby resulting in a reduction from over 67,000 to ~46,000 compounds. Tier II applied the homologous structure classifier which resulted in a reduction from 46,000 to 38 compounds, including *α*-cristobalite SiO_2_. In the final step, Tier III, DFT calculations of the full elastic tensors identified 8 structures (*α*-cristobalite included) predicted to exhibit negative homogeneous Poisson’s ratios

To date, the Materials Project^31^ contains data for over 67,000 distinct inorganic crystalline materials where of over 4000 have an associated calculated elastic tensor. Benchmarks of the Materials Project elastic tensor workflow suggest that it reproduces elastic-tensor derived bulk and shear moduli well; within 15% of experimentally measured values. However, the workflow requires computationally demanding input parameters to ensure this level of accuracy^25^. It is currently projected to take years to calculate the elastic tensor for all known crystalline compounds using available supercomputing resources. Hence, approaches which prioritize certain compounds based on structure-property descriptors are useful to accelerate the rate of discovery. For example, predictive statistical learning methods have been used to estimate bulk and shear moduli of materials in the MP database, illustrating that existing DFT-based data on elasticity and structure can be leveraged to guide the search for specific elastic behavior^33^. In this work, to expedite the discovery process with respect to Poisson’s ratio, materials were screened though a set of criteria designed to reduce the number of elasticity calculations required to identify novel auxetics. In Tier I (see Fig. 2), we first narrowed our candidate pool by applying a stability criterion, retaining only materials for which the formation energy is less than 100 meV per atom above the convex hull. This removes materials from consideration that may be challenging to synthesize^34^. From this set, computationally tractable materials with fewer than 50 sites per unit cell were retained, resulting in roughly 46,000 structures (see Fig. 2, Tier I). We note that this constraint necessarily excludes many zeolites and porous materials, which may exhibit negative Poisson’s ratios^8, 20^. However, auxetic behavior in those materials may be due to structural motifs at a larger scale than those targeted in our search^16^.

In Tier II, we used a homologous structure motif to identify materials likely to exhibit auxetic behavior using *α*-cristobalite as a structural archetype. The advantage of homologous structure matching over methods using select geometric descriptors is that information with seemingly inconspicuous significance is inherently included in the crystallographic description of the structure. This is especially relevant for inorganic crystalline auxetic materials because so few positive examples exist. To implement the structural screening, we utilized the pymatgen structure matcher^32^ to construct a structure similarity classifier which compared the roughly 46,000 materials that passed the first tier to *α*-cristobalite’s corner-connected tetrahedral motif. A species-agnostic algorithm was used to initialize the structure matcher in order to expand the scope of the search beyond binary chemical systems.

The result of the overall screening process was a reduction in the number of candidate materials for full DFT elasticity calculations by three orders of magnitude, from more than 67 000 materials to 38. We note that this set included the known auxetic *α*-cristobalite SiO_2_. The Poisson’s ratios of the candidate compounds were subsequently calculated using the Materials Project’s high-throughput DFT elasticity workflow, which has been benchmarked to within 15% of available experimental measurements^25^. Of the 38 elastic tensor calculations, 30 successfully converged, passing all of the built-in filters of the workflow. These filters include removing materials that exhibit negative eigenvalues of the elastic tensor, which indicates soft modes and mechanical/dynamic instabilities at low temperatures. The average, homogeneous Poisson’s ratio of the set was 0.07 and an average negative Poisson’s ratio was obtained for 8 out of the 30 (Supplementary Table 1). In addition to *α*-cristobalite, four polymorphs of SiO_2_, two ternary metal vanadates, and a high-temperature phase of AlPO_4_ were predicted to exhibit negative homogeneous Poisson’s ratios. Another 5 materials of the 30 exhibited positive but near-zero homogeneous Poisson’s ratios. (Table 1).

**Table 1:** Predicted directional and homogeneous Poisson’s ratios for a selection of Tier 2 materials

Material	Structure	Space group	Prev. synthesized?	Directional Poisson		Homo. Poisson (*μ*)	**Class**
				(*ν*_min_)	(*ν*_max_)		
SiO_2_ (*α*C)	Tetragonal	*P*4_1_2_1_2 [92]	Yes^12^	−0.580	−0.030	−0.29	Auxetic
HT-AlPO_4_	Orthorhombic	*C*222_1_ [20]	Yes^18^	−0.582	−0.043	−0.28	Auxetic
SiO_2_ (a)	Tetragonal	*P*4_3_2_1_2 [96]	No	−0.551	−0.029	−0.27	Auxetic
SiO_2_ (b)	Orthorhombic	*P*2_1_2_1_2_1_ [19]	No	−0.518	0.027	−0.20	Auxetic
SiO_2_ (c)	Orthorhombic	*P*2_1_nb [33]	No	−0.478	0.168	−0.05	Anepirretic
FeV_3_O_8_	Triclinic	*P*1 [1]	No	−0.379	0.168	−0.05	Anepirretic
CoV_3_O_8_	Triclinic	*P*1 [1]	No	−0.452	0.293	−0.04	Anepirretic
SiO_2_ (d)	Monoclinic	*C*2/*c* [15]	No	−0.651	0.403	−0.01	Anepirretic
FePO_4_	Orthorhombic	*Pn*2_1_ *a* [33]	No	−0.328	0.269	0.01	Anepirretic
SiO_2_ (*β*C)	Orthorhombic	*I* $\bar 4$2*d* [122]	Yes^43^	−0.102	0.027	0.05	Anepirretic
BVO_4_	Tetragonal	*I* $\bar 4$ [82]	No	−0.502	0.392	0.06	Anepirretic
GaPO_4_	Orthorhombic	*C*222_1_ [20]	Yes^42^	−0.343	0.548	0.07	Anepirretic
MnV_3_O_8_	Triclinic	*P*1 [1]	No	−0.338	0.492	0.09	Anepirretic
GeO_2_	Tetragonal	*P*4_1_2_1_2 [92]	Yes^51^	−0.301	0.448	0.10^+^	Meiotic

**Fig. 3:**
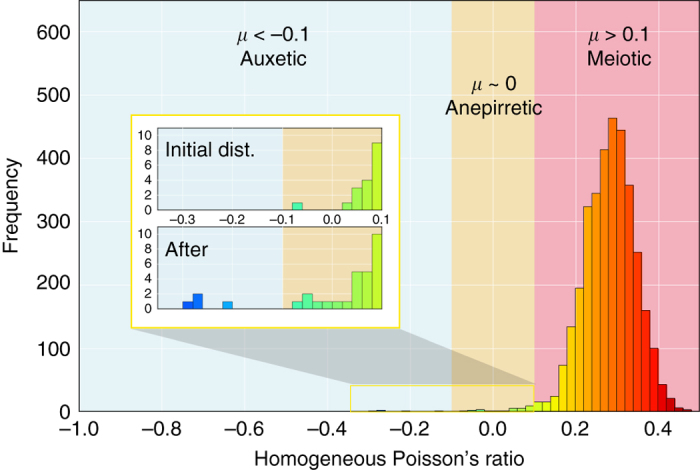
The distribution of homogeneous Poisson’s ratio. Within the set of materials with elasticity data in the Materials Project database, meiotic materials far outnumber auxetic and anepirretic materials, which make up less than 1% of the total distribution. The inset shows the number of auxetic and anepirretic materials before and after the homologous structure search based on *α*-cristobalite SiO_2_

Instead of classifying the materials solely by the sign of the Poisson’s ratio, we introduce a new criterion based on both sign and magnitude of *μ*. Following the precedent set by Evans in assigning materials by Poisson’s ratio under Greek terms^1^, we have grouped structures with |*μ*| ≤ 0.1 separately from homogeneously auxetic materials. We denote this class of materials ‘anepirretic’ after the Greek word ανεπηρε′αστος, which means “unaffected”. These materials will maintain a nearly constant cross-section under linear-elastic tensile strain. The remaining, bulk majority of materials with *μ* > 0.1 are denoted ‘meiotic’ after the corresponding antonym, μειωτικóς. Figure 3 shows the distribution of computed homogeneous Poisson’s ratios in the Materials Project, which comprises the largest elastic tensor resource to date^25^. While the overwhelming majority (99%) of materials display meiotic behavior, the current search identified a statistically significant number of calculated negative or near-zero homogeneous Poisson’s ratios. Of the 30 materials that passed Tier I and II, 1 known auxetic, 3 new auxetic, 9 new anepirretic, and 17 meiotic Poisson’s ratios were predicted. This has greatly increased the total number of predicted auxetic and anepirretic materials in the MP database, as shown in the inset of Fig. 3. In the following section we discuss the resulting materials with negative Poisson’s ratios (auxetic and anepirretic) in more detail.

### Resulting auxetic and anepirretic materials

The results of the elastic analysis for each of the auxetic and anepirretic materials identified by the screening process are included in Table 1. Also included in Table 1, due to its pertinence to the role of chemistry in controlling the Poisson’s ratio of materials, is GeO_2_, a meiotic compound exhibiting a structure identical to *α*-cristobalite.

Two novel silica polymorphs, denoted SiO_2_(a) and (b), were identified as auxetic by the screening process. We note that SiO_2_(a) is the enantiomer of *α*-cristobalite SiO_2_, hence it is not surprising that its elastic properties are close to those of its chiral partner. Coh and Vanderbilt investigated the structural stability of cristobalite and described how the *α* and *β* structures may be considered higher-symmetry special cases of a three-dimensional manifold with general *P*2_1_2_1_2_1_ symmetry accessed by RUMs^19^. Both SiO_2_(a) and (b) appear on this manifold along with *α* and *β* cristobalite. Intuitively, the homogeneous Poisson’s ratios of these structures fall between those of *α* and *β*-cristobalite SiO_2_.

Two other silica polymorphs, denoted SiO_2_(c) and (d), as well as *β*-cristobalite, denoted SiO_2_(*β*C), were calculated as anepirretic. Furthermore, two ternary metal vanadates; FeV_3_O_8_ and CoV_3_O_8_ were identified as anepirretic with predicted Poisson’s ratios of −0.05 and −0.04, respectively. The four silica polymorphs SiO_2_ (a)–(d) and the two ternary vandates are part of the growing number of materials in the Materials Project which are ‘hypothetical’ new compounds^31, 35^, e.g., they have been generated using various transformation schemes, such as substitutions on existing materials, to explore novel chemistries and structures for different applications. Hence, there is no existing literature on synthesis and testing of these materials; however, they exhibit favorable formation energies and may be amenable for synthesis^34^.

The potentially most interesting of the novel identified auxetic materials is a high-temperature phase of aluminum orthophosphate: HT-AlPO_4_. This known polymorph exhibits a structure very similar to *α*-cristobalite but has a lower crystal symmetry (C222_1_) due to its stoichiometry. Our calculations predict it has a Poisson’s ratio of −0.28, rivaling that of *α*-cristobalite SiO_2_. Berlinite AlPO_4_ spontaneously transforms to HT-AlPO_4_ at elevated temperatures, which remains metastable at room temperature^18, 36^. Although NAT-type aluminophosphate zeolites have been investigated for negative Poisson’s ratio^37, 38^, to our knowledge HT-AlPO_4_ has not been experimentally investigated for auxetic behavior^39^.

**Fig. 4:**
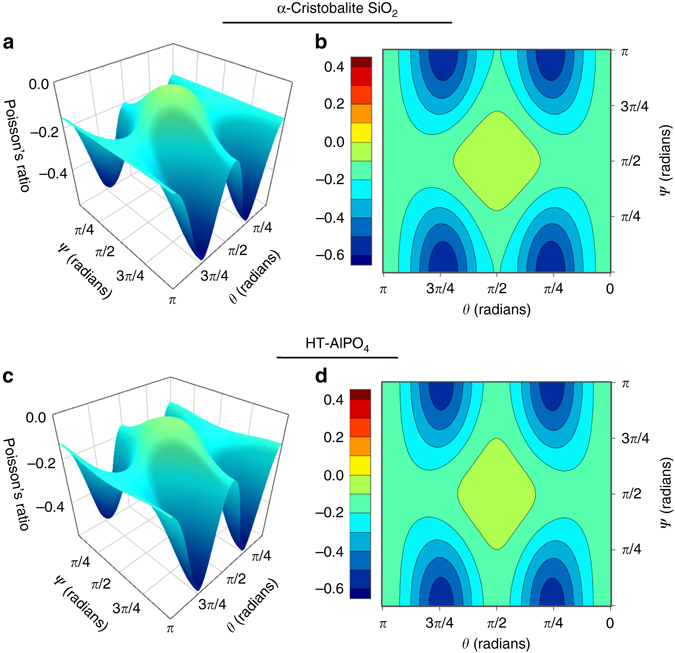
Directional Poisson’s ratio maps for *α*-cristobalite SiO_2_ and HT-AlPO_4_. *ν*_32_ was calculated from the elastic tensors of for *α*-cristobalite SiO_2_ **a**, **b** and HT-AlPO_4_ **c**, **d** under basis rotations around the Eulerian *z*, *x*ʹ, and *z*ʹʹ axes. These rotations were: *ϕ* = *π*/4, *θ* = [0, *π*], and *ψ* = [0, *π*] for *α*-cristobalite and *ϕ* = 0, *θ* = [0, *π*], and *ψ* = [0, *π*] for HT-AlPO_4_

In order to study the extremal values of the anisotropic directional Poisson’s ratio, *ν*_*ij*_, for the materials identified in Tier 3, we calculate *ν*_32_ over the entire range of possible orientations and compare to the known *α*-cristobalite SiO_2_. The elastic tensors were rotated around the Eulerian *z*, *x*ʹ, and *z*ʹʹ directions between 0 and 2*π* radians at a resolution of *π*/30 and for each orientation, *ν*_32_ was recorded as a function of the rotation angles. Figure 4 shows the variation in the anisotropic directional Poisson’s ratio *ν*_32_ for *α*-cristobalite and HT-AlPO_4_ over the subspace of orientations spanned by rotations about the *x*ʹ and *z*ʹʹ axes. In order to more directly compare the Poisson response of the two materials, the elastic tensor of *α*-cristobalite was first rotated by *π*/4 around *z* to align its orientation with that of HT-AlPO_4_’s elastic tensor with respect to its conventional standard unit cell.

**Fig. 5:**
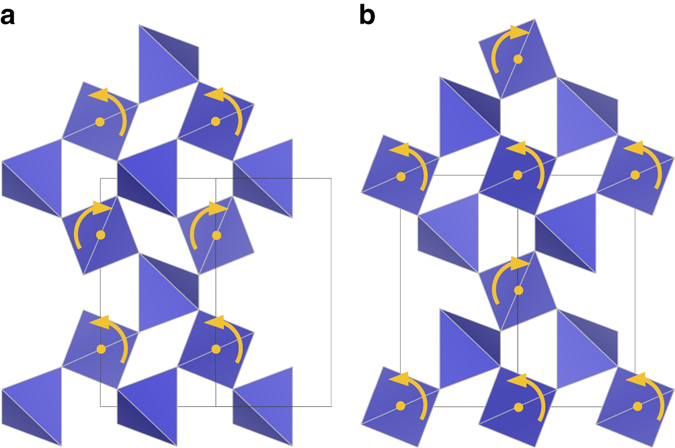
Tetrahedral rotations in *α*-cristobalite SiO_2_. Illustration of the rotational axes for the SiO_4_ tetrahedra under tensile strain along the [001] direction as viewed from the **a** [110] direction and the **b** [$\bar 1$10] direction in *α*-cristobalite SiO_2_

**Fig. 6:**
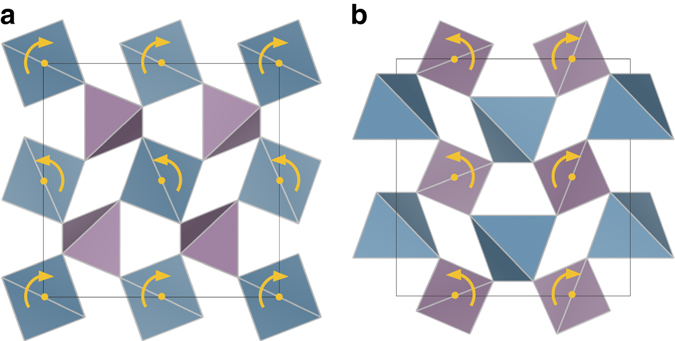
Tetrahedral rotations in HT-AlPO_4_. Illustration of rotational axes of AlO_4_ and PO_4_ tetrahedra under tensile strain in the [001] direction. In **a** the rotational axis of the AlO_4_ tetrahedra is depicted as viewed from the [100] direction whereas **b** shows the rotational axis of the PO_4_ tetrahedra as viewed from the [010] direction

Indeed, *ν*_max_ is predicted as negative for both *α*-cristobalite and HT-AlPO_4_, implying that, similar to *α*-cristobalite SiO_2_, HT-AlPO_4_ will behave auxetically when strained in any direction. We note that these results are consistent with other DFT calculations of the elastic properties of *α*-cristobalite, which slightly overestimate the auxetic response such that *ν*_max_ < 0, whereas Brillioun spectroscopy indicates *ν*_max_ = +0.08^40^. Nevertheless, *α*-cristobalite SiO_2_ still exhibits overall auxetic behavior in experiment^12^ and the same is expected for HT-AlPO_4_. We find that *ν*_min_ appears on the *x*ʹ–*z*ʹʹ rotation surfaces of *α*-cristobalite and HT-AlPO_4_ at coinciding rotations, hence the character of their Poisson behavior is highly similar. Figures 5 and 6 illustrate the tetrahedral rotations of the two materials for [001] tensile strains, which are also congruent in character. These predictions for RUM rotations under strain are consistent with the results of previous investigations into the origins of auxetic behavior in *α*-cristobalite^14, 41^. Moreover, these soft rotational modes are also activated in the *α*–*β* transformation of cristobalite SiO_2_^18^. SiO_2_ (a) also exhibits a negative *ν*_max_, and hence global auxetic behavior, and SiO_2_ (b) manifests a *ν*_max_ very close to 0.0, indicating auxetic behavior in a majority of directions.

Auxetic materials have conventionally been classified as “partially auxetic” if they possessed one or more negative directional Poisson’s ratios or “completely auxetic” if they exhibited no positive values for *ν*_*ij*_^22^. While completely auxetic materials will always be homogeneously auxetic, partially auxetic materials can average to auxetic, anepirretic, or meiotic depending on the distribution of *ν*_*ij*_. Anepirretic materials typically exhibit directional Poisson’s ratios that are distributed across both positive and negative values resulting in a homogeneous Poisson’s ratio close to zero. As indicated in Table 1, a number of the final candidates display positive but near-zero homogeneous Poisson’s ratios. The SiO_2_ (c) polymorph as well as the ternary vandates display weakly negative Poisson’s ratios while positive *ν*_max_. Uniformly small directional Poisson’s ratios as well as combinations of positive and negative directions can minimize the magnitude of the homogeneous Poisson’s ratio in anepirretic materials, which may be a useful materials design feature for applications where transverse strain is undesirable.

## Discussion

Accelerated discovery of novel materials with target properties is desirable to meet current and future material needs. In this study, we show how a tiered search, targeting specific structural motifs, can greatly accelerate the discovery of materials with unusual elastic behavior.

To date, very few inorganic crystalline materials other than *α*-cristobalite SiO_2_ are known to exhibit homogeneous auxetic behavior. Prior to this study, through the Materials Project calculations of elastic tensors, a tungsten oxide (WO_3_) with weakly negative Poisson’s ratio, *μ* = −0.08, was serendipitously found among the first 2000 materials calculated^25^. This result represents an ~0.02% chance to discover a material with a negative homogeneous Poisson’s ratio. Using the target screening described here, we find that almost one in five of the materials identified by the homologous structure search exhibit negative homogeneous Poisson’s ratios and 3/30 are proposed new homogeneous auxetics. Hence, the approach delivers an improvement of three orders of magnitude.

It is important to note that the Materials Project database does not discern between materials that have been synthesized and those that remain hypothetical. Previously synthesized or known materials, identified by the tiered search as either auxetic or anepirretic, are HT-AlPO_4_, *β*-cristobalite SiO_2_, and GaPO_4_. Both phosphates are metastable at room temperature and can be flux-grown or synthesized through annealing of quartz-structured AlPO_4_ and GaPO_4_ above the cristobalite transition temperature with subsequent quenching^36, 42^. However, *β*-cristobalite SiO_2_ spontaneously transforms to *α*-cristobalite upon cooling and must be chemically stabilized to exist at room temperature^43^. The remaining seven materials identified by the search as auxetic or anepirretic are hypothetical compounds with low formation energies which indicate a fair chance of successful synthesis^34^. The polymorphs SiO_2_ (a), (c), and (d) are frameworks generated by simulated annealing^35^ while SiO_2_ (b) represents an early suggestion for the *α*-cristobalite structure proposed in 1932. The four vanadates (FeV_3_O_8_, CoV_3_O_8_, MnV_3_O_8_, and BVO_4_) originate from a variety of structure manipulations of known compounds, such as orderings of disordered structures, and cation substitution in such frameworks^44^. We stress that the fact that these materials are hypothetical does not necessarily preclude the possibility that they can be made, and identifying them as auxetic may inspire design and discovery of similar compounds. Additionally, we note that the restriction on energy above the convex hull in Tier I of the screening process does not reflect an absolute limit for stability. As such, this screening method may exclude materials that can be synthesized and exploring higher energies may lead to the discovery of additional homogeneously auxetic materials of interest.

**Fig. 7:**
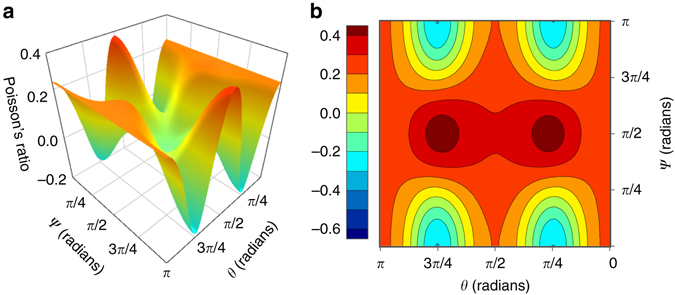
Directional Poisson’s ratio map for *α*-cristobalite GeO_2_. The Poisson’s ratio surface **a** and contour plot **b** for *α*-cristobalite GeO_2_ show the variation of *ν*_32_ as derived from the calculated elastic tensor under basis rotations around the Eulerian *z*, *x*ʹ, and *z*ʹʹ axes. These rotations were: *ϕ*  = *π*/4, *θ* = [0, *π*], and *ψ* = [0, *π*]

The presented work is built on the hypothesis that the structural motif—encompassed by rigid, corner-connected tetrahedra and open structures—is the dominating driver for auxetic or anepirretic behavior. As such, our search was intentionally agnostic to chemistry. While this hypothesis is clearly validated by the high success rate of the Tier III materials, chemistry also clearly plays a role in differentiating the Poisson’s ratios of materials with similar structures. As an example, we offer *α*-cristobalite GeO_2_ which exhibits the exact same crystal structure, symmetry, and stoichiometry as *α*-cristobalite SiO_2_. Indeed, GeO_2_ has previously been proposed as an auxetic material^9^. However, while auxetic directions are found in this material (see Fig. 7), the majority of strain directions result in positive Poisson’s ratios and hence, its homogeneous Poisson’s ratio is calculated as 0.10. Thus, we find that the compound most similar to alpha-cristobalite both in structure and chemistry is meiotic. This example is similarly mirrored by HT-AlPO_4_ being auxetic while the highly related compound GaPO_4_ is not. We speculate that small differences in bonding and bond lengths may be enough to influence the soft rotational modes that underlie auxetic behavior. Hence, this particular structural motif is found to be a highly correlated, but insufficient, descriptor for auxetic behavior. We speculate that additional or broader structural motifs could be employed as markers for potential auxetic and anepirretic behavior.

In summary, using a structure-matching procedure, together with stability and computational cost screening, drastically reduced the number of DFT calculations required to predict three previously unknown homogeneously auxetic compounds, whereof one is a known material; the high-temperature AlPO_4_. Furthermore, our screening identified nine anepirretic materials, which are characterized by possessing a combination of both auxetic and meiotic directions resulting in an almost zero Poisson’s ratio. The success of our approach suggests that negative or near-zero Poisson’s ratio is a common feature of materials with cristobalite-like structures, such that a dramatically higher success rate is achieved if the structure motif is included in a materials screening. However, we also note that chemistry is not unimportant as several *α*-cristobalite-structured materials still exhibit meiotic behavior. We hope that the results of this study demonstrate the advantage of combining materials databases and intelligent materials design to accelerate materials discovery.

## Methods

### Ab initio calculations

The high-throughput methodology and workflow used to calculate the elastic tensors was documented and benchmarked for all experimentally known elastic tensors as described previously in detail by de Jong et al.^25^ In short, the elastic workflow begins with a structure optimization and subsequently calculates the stress of independent deformations of the optimized structure. Independent linear and shear strains between −1 and 1% are applied to the relaxed structures and the resultant stress tensors are calculated for each of these strain states. The calculations use first-principles DFT as implemented in the VASP code^45, 46^. Because the calculations require converged determinations of the bulk stress, a dense mesh of 7000 k-points per reciprocal atom and a cutoff energy of 700 eV for the plane-wave basis set are used. Exchange-correlation effects of the electronic density are accounted for using the Perdew–Burke–Ernzerhof functional^47^. In addition, standard Materials Project Hubbard U corrections are used for a number of transition metal oxides^48^, as documented and implemented in the pymatgen VASP input sets^32^. Resulting elastic tensors are filtered on the basis of multiple criteria for mechanical stability and reliability as previously documented^25^. The full content of the workflow and analytical tools used to generate this and all other MP elastic tensor data may be found in the MPWorks and pymatgen code packages at github.com/materialsproject.

### Calculation of Poisson’s ratios

The homogeneous approximation of the Poisson’s ratio was calculated from the elements of the calculated elastic tensors as:

1 $$\mu = \frac{{3{K_{VRH}} - 2{G_{VRH}}}}{{6{K_{VRH}} + 2{G_{VRH}}}}$$

where *K*_*VRH*_ and *G*_*VRH*_ are the Voigt–Reuss–Hill averaged bulk and shear moduli, respectively^25^. Furthermore, the anisotropic Poisson response of materials were investigated through the calculation of the directional Poisson ratios, *ν*_*ij*_, determined from the coefficients of the Voigt compliance tensor (inverse of the elastic tensor), *S*, as:

2 $${\nu _{ij}} = - \frac{{{S_{ij}}}}{{{S_{ii}}}}$$

where the value for *ν*_*ij*_ represents the ratio of deformation in the principal direction *j* to a uniaxial strain applied along the orthogonal principal direction *i* in the basis frame of the elastic tensor^26^. The global minimum and maximum directional Poisson’s ratios were found by rotating the original elastic tensor through the range of possible orientations via the Euler–Rodriguez and Cayley method and Euler angle rotations around the *z*, *x*ʹ, and *z*ʹʹ axes at a resolution of *π*/30 radians^49^.

### Determination of tetrahedral rotations

The tetrahedral rotations displayed in Figs. 5 and 6 were derived from ideal tensile strain calculations^50^, in which the equilibrium bulk structures are deformed in a single direction and optimized isotropically in the perpendicular directions. This allowed for an estimate of the structural response to a uniaxial load, and further reinforces the prediction of auxetic behavior in elastic tensor results.

### Structure comparisons

The structure-comparison algorithm described in this study was implemented using the structure matcher in Pymatgen^32^. Structure-matching proceeds by: 1. reducing to unit cells and comparing the total number of sites, 2. further reducing to Niggli cells and scaling by volume, 3. removing oxidation states, and 4. comparing, within specified tolerances, the lattice angles and atomic positions of all permutations of valid lattices. A full description of the algorithm is provided. (Supplementary Methods) The structure matcher was initialized with a framework comparator, which ignores species and oxidation state when comparing sites between two structures, and the following structure-matching parameters: fractional length tolerance = 0.2, fraction of average free length per atom = 0.5, and angle tolerance = 5°.

### Data availability

The code and data used to develop the screening process in this study are available at http://pymatgen.org and https://materialsproject.org. Any other information supporting the conclusions presented is available from the authors upon request.

## Electronic supplementary material

Supplementary Information

Peer Review File
