# Supplementary material for: Computational prediction of new auxetic materials
Source: Nat Commun. 2017 Aug 22;8:323. doi: 10.1038/s41467-017-00399-6 (PMC5567361; doi:10.1038/s41467-017-00399-6)
Supplement: Supplementary file 1 — Supplementary Information [file 41467_2017_399_MOESM1_ESM.pdf]

File Name: Supplementary Information
Description: Supplementary Table and Supplementary Methods

File Name: Peer Review File
Description:

Supplementary Table 1. Tier II materials with successful elasticity calculations.

Material	MP-ID	Space Group	E Above Hull (eV/atom)	ν_{min}	ν_{max}	μ
$BAsO_4$	mp-3277	$I\bar{4}$ (82)	0.000	-0.34	0.49	0.15
BPO_4	mp-3589	$I\bar{4}$ (82)	0.000	-0.34	0.39	0.12
PNO	mp-36066	Cc (9)	0.000	-0.36	0.41	0.12
$AlPO_4$	mp-4051	$C222_1$ (20)	0.002	-0.58	-0.04	-0.28
SiO_2	mp-546794	$I\bar{4}2d$ (122)	0.000	-0.47	0.36	0.05
$GaPO_4$	mp-553932	$C222_1$ (20)	0.007	-0.35	0.46	0.07
SiO_2	mp-554089	$Pna2_1$ (33)	0.003	-0.48	0.21	-0.05
SiO_2	mp-556961	$P2_12_12_1$ (19)	0.005	-0.52	0.05	-0.20
$GaPO_4$	mp-677335	$I\bar{4}$ (82)	0.000	-0.34	0.51	0.20
SiO_2	mp-6945	$P4_12_12$ (92)	0.003	-0.57	-0.03	-0.29
SiO_2	mp-7029	$P4_32_12$ (96)	0.003	-0.55	-0.03	-0.26
PNO	mp-753671	$I2_12_12_1$ (24)	0.000	-0.36	0.45	0.12
WN_2	mp-754628	$Pna2_1$ (33)	0.058	0.22	0.57	0.40
$FePO_4$	mp-764338	$I\bar{4}$ (82)	0.002	-0.38	0.46	0.18
VBO_4	mp-778780	$I\bar{4}$ (82)	0.000	-0.50	0.38	0.06
GeO_2	mp-7812	$P4_12_12$ (92)	0.001	-0.30	0.44	0.10
$AlPO_4$	mp-7848	$I\bar{4}$ (82)	0.000	-0.40	0.39	0.13
$AlAsO_4$	mp-7849	$I\bar{4}$ (82)	0.000	-0.34	0.50	0.18
$FePO_4$	mp-540111	$Pna2_1$ (33)	0.000	-0.33	0.26	0.01
$CoPO_4$	mp-689940	$Pna2_1$ (33)	0.006	-0.31	0.53	0.17
SiO_2	mp-557837	C2/c (15)	0.006	-0.66	0.48	-0.01
SiO_2	mp-8352	$Fd\bar{3}m$ (227)	0.012	-0.07	0.73	0.34
GeS_2	mp-7582	$I\bar{4}2d$ (122)	0.021	-0.29	0.67	0.23
PNO	mp-36779	P1 (1)	0.025	-0.39	0.44	0.11
V_3CoO_8	mp-766784	P1 (1)	0.037	-0.46	0.30	-0.04
$ZnSO_4$	mp-545756	$F\bar{4}3m$ (216)	0.038	-0.23	1.12	0.44
SiS_2	mp-7583	$I\bar{4}2d$ (122)	0.052	-0.35	0.64	0.19
V_3MnO_8	mp-776985	P1 (1)	0.057	-0.33	0.54	0.09
V_3FeO_8	mp-775001	P1 (1)	0.080	-0.41	0.24	-0.05
$BePO_4$	mp-760410	$I\bar{4}$ (82)	0.067	-0.58	0.74	0.12

Supplementary Methods

Pymatgen structure matcher algorithm.

Parameters:

ltol: fractional length tolerance = 0.2
stol: fraction of average free length per atom = 0.5
angle_tol: angle tolerance = 5°

1. Given two structures: s1 and s2
2. Reduce to primitive cells.
3. If the number of sites do not match, *return False*.
4. Reduce to s1 and s2 to Niggli Cells.
5. Scale s1 and s2 to same volume.
6. Remove oxidation states associated with sites.
7. Find all possible lattice vectors for s2 within shell of ltol.
8. For s1, translate an atom in the smallest set to the origin.
9. For s2: find all valid lattices from permutations of the list of lattice vectors (invalid if: det(Lattice Matrix) < half volume of original s2 lattice.)

For each valid lattice:

1. If the lattice angles of are within tolerance of s1, basis change s2 into new lattice.
2. For each atom in the smallest set of s2:
    I. Translate to origin and compare fractional sites in structure within a fractional tolerance.
    II. If true:
        a. Convert both lattices to Cartesian and place both structures on an average lattice.
        b. Compute and return the average and max rms displacement between the two structures normalized by the average free length per atom.
        c. if fit function called:
            i. if normalized max rms displacement is less than stol *return True*
        d. if get_rms_dist function called:
            i. if normalized average rms displacement is less than the stored rms displacement, store and continue. (This function will search all possible lattices for the smallest average rms displacement between the two structures.)
